# Transgressive incidents targeted on staff in forensic psychiatric healthcare: a latent class analysis

**DOI:** 10.3389/fpsyt.2024.1394535

**Published:** 2024-05-20

**Authors:** Iris Frowijn, Erik Masthoff, Jeroen K. Vermunt, Stefan Bogaerts

**Affiliations:** ^1^Department of Developmental Psychology, Tilburg University, Tilburg, Netherlands; ^2^Fivoor Science and Treatment Innovation (FARID), Rotterdam, Netherlands; ^3^Department of Methodology and Statistics, Tilburg University, Tilburg, Netherlands

**Keywords:** aggression, forensic psychiatry, mental health workers, forensic patients, transgressive incidents, latent class analysis

## Abstract

Transgressive incidents directed at staff by forensic patients occur frequently, leading to detrimental psychological and physical harm, underscoring urgency of preventive measures. These incidents, emerging within therapeutic relationships, involve complex interactions between patient and staff behavior. This study aims to identify clusters of transgressive incidents based on incident characteristics such as impact, severity, (presumed) cause, type of aggression, and consequences, using latent class analysis (LCA). Additionally, variations in incident clusters based on staff, patient, and context characteristics were investigated. A total of 1,184 transgressive incidents, reported by staff and targeted at staff by patients between 2018-2022, were extracted from a digital incident reporting system at Fivoor, a Dutch forensic psychiatric healthcare organisation. Latent Class Analysis revealed six incident classes: 1) *verbal aggression with low impact*; 2) *verbal aggression with medium impact*; 3) *physical aggression with medium impact*; 4) *verbal menacing/aggression with medium impact*; 5) *physical aggression with high impact*; and 6) *verbal and physical menacing/aggression with high impact*. Significant differences in age and gender of both staff and patients, staff function, and patient diagnoses were observed among these classes. Incidents with higher impact were more prevalent in high security clinics, while lower-impact incidents were more common in clinics for patients with intellectual disabilities. Despite limitations like missing information, tailored prevention approaches are needed due to varying types of transgressive incidents across patients, staff, and units.

## 1 Introduction

In 2020, 82% of mental healthcare professionals in the Netherlands, including those in forensic psychiatry, reported encountering transgressive behavior in patients interactions (1). Verbal aggression was the most common form of transgressive behavior by patients towards staff, accounting for 79% of reported incidents (1). Generally, transgressive behavior encompasses actions that violate personal boundaries (2), and can manifest as verbal aggression, (sexual) intimidation, discrimination, bullying, and physical aggression. In this study, transgressive behavior is defined as patient aggression directed towards professionals working in the forensic field. As a direct result of transgressive incidents, staff mostly reported feeling upset (38%), and mental (8%) and physical (5%) problems (1). Long-term consequences may include increased burnout rates, as shown in forensic mental healthcare workers (3–5). Apart from adverse consequences for healthcare workers, transgressive behavior can hinder therapeutic progress of the patient, potentially prolonging treatment due to disruptions in the clinical atmosphere (e.g., revocation of privileges) and/or the induction of fear and stress among professionals (6, 7). Hence, preventive measures are needed (1, 8).

Transgressive incidents often stem from aggression regulation problems that are common among forensic psychiatric patients, which can be reasons for receiving (mandatory) forensic treatment (9, 10). According to the General Aggression Model (GAM; 11), aggressive behavior arises from a combination of person-specific and situational factors influencing cognitive, affective, and arousal processes, consequently influencing appraisal and decision-making preceding aggressive incidents. Forensic patients often have hostile world views and perceive aggression positively, using it as a coping mechanism (12). Mandatory forensic treatment aims to address aggressive behavior and its underlying causes, thereby mitigating individual risk factors for (violent) recidivism and ultimately enabling reintegration into society, following the principles of the Risk-Need-Responsivity (RNR) model (13, 14).

However, not all forensic psychiatric patients have the same risk factors for recidivism, as they constitute a heterogeneous group with diverse characteristics and psychopathologies (15). Patient psychopathology may influence aggression, with pathways to aggressive behavior varying depending on psychopathology (16, 17). Dynamic risk factors for inpatient aggression exhibit different patterns across different patient groups (15, 18, 19). For instance, aggression in schizophrenic patients often results from active psychotic symptoms and is reactive (e.g., following the denial of a request) (9), while patients with personality disorders may engage in aggression more frequently for instrumental purposes (e.g., to obtain tangibles) (20). Hence, the types of transgressive behavior might show different characteristics as well, which is why there is a need to explore what types of transgressive behavior are shown by which patients towards which staff members.

Forensic mental healthcare workers bear a dual responsibility, balancing between the patient’s therapeutic needs with legal mandates, posing ethical and practical dilemmas (21). Consequently, these dilemmas can result in transgressive behaviors (by patients), which can have both physical and psychological consequences for staff (22). While physical consequences are usually overt (i.e., physical injuries), the psychological consequences often remain covert, encompassing post-traumatic stress symptoms, feelings of guilt, shame, and self-blame (23). Moreover, prolonged exposure to less severe forms of violence, common in forensic mental healthcare settings, is associated with higher rates of psychological distress (24). A qualitative study in forensic mental health staff revealed that the severity of incidents alone does not determine its impact on staff members. Rather, their appraisal and attitude toward the patient and the incident play significant roles (25). Especially attitudes towards patient psychopathology and the role of the latter in the incident’s manifestation are related to the emotional responses of staff. For instance, aggressive incidents involving patients diagnosed with schizophrenia were found to have a lesser impact on staff than similar incidents with other patient groups, resulting in different emotional responses (25).

The occurrence of transgressive incidents may also depend on other staff characteristics, such as age and experience. A systematic review revealed that younger and less experienced staff are generally more susceptible to workplace violence compared to their older and more experienced counterparts (26). However, findings in this regard are mixed, for instance, in a Veterans Administration neuropsychiatric hospital, more experienced nurses faced more assaults, especially from long-term patients (27). Conversely, younger staff members (< 40 years old) reported fewer physically aggressive incidents from patients, while young female staff members were more frequent targets of sexual harassment (28). In a US forensic psychiatric hospital, work experience was not significantly correlated with assaults rates (29). Instead, they found that men (vs. women) and working in the ward (vs. clinical and supervisory staff) were associated with higher risks of physically aggressive incidents, depending on the worker’s stress reactivity (29). Additionally, younger and less experienced staff exhibited more burnout symptoms, including emotional exhaustion and depersonalization, compared to their older, more experienced colleagues (30). These findings indicate the nuanced relationship between age, work experience, and the risks and consequences of transgressive behavior and workplace aggression, highlighting the influence of individual characteristics among workers.

From a patient perspective, individual patient characteristics can influence the occurrence of incidents. For instance, a meta-analysis found factors such as younger age, male, and a diagnosis of schizophrenia as contributors to increased aggressive incidents (31). Younger (vs. older) forensic patients were more susceptible to severe incidents (32), with males being more frequently involved in or responsible for incidents than females (33). However, a small group of patients accounted for most incidents displayed by female forensic patients, and severe incidents causing physical injuries were rare among patients (33). Studies further found that male patients were more likely to direct violence towards others, specifically fellow patients, while female patients were more likely to target themselves or staff (34, 35). A Dutch study found that female forensic patients were responsible for a higher number of incidents compared to male forensic patients (32). This difference can be attributed to psychopathological factors (36, 37), as female forensic patients often present with more complex psychopathology, particularly borderline personality disorder and impulsivity, which account for their association with physically aggressive incidents (36, 37).

### 1.1 The present study

This study aims to investigate transgressive behavior within forensic psychiatric settings and its impact on staff. Reported transgressive incidents will be evaluated to identify clusters using latent class analysis (LCA). Factors considered include the extent of impact, severity, type of aggression, consequences, cause, and target of the transgressive behavior, all from the perspective of the reporting staff member. As prior studies on incident classification using these criteria are lacking, our approach is exploratory, without specific hypotheses for the analysis of incident clusters. In the next step, rather than exclusively investigating patient characteristics (e.g., 35) or staff characteristics (e.g., 29), we aimed to explore whether specific incidents vary between patients and staff groups. We will consider various characteristics, such as age, gender, staff function, and patient psychopathologies. These findings may lead to targeted psycho-educational interventions, such as aggression-prevention training for both staff and patients. It is hypothesized that men (vs. women) and nursing staff (vs. clinical and managing staff), will be more susceptible to severe and physical aggressive incidents (29). Patients diagnosed with schizophrenia and younger individuals are expected to be associated with more severe and physical aggressive incidents (31). Furthermore, incidents involving patients with personality disorders are likely to have a more pronounced impact on staff members (25).

## 2 Method

### 2.1 Participants and procedure

This study used existing data obtained from a digital system within Fivoor, an organization specializing in forensic and intensive psychiatric care in the Netherlands, with 28 locations. These include inpatient centers with varying security levels, outpatient settings, flexible assertive community treatment (FACT), probation services, material legal services, and specialized settings for patients with mental disabilities (for more information see: https://fivoor.nl). With approximately 1,700 staff members taking care of about 8,000 patients, all incidents are mandatory for reporting via the digital system known as “Veilig Incident Melden (Secure Incident Reporting)”. During the period 2018-2022, a total of 11,487 transgressive incidents were reported. Although this information is routinely used for internal evaluations, it has not been used for research goals until now. For the current study, the data was anonymized to ensure that reported incidents could not be traced back to individual workers or patients. Given that pre-collected data was used, informed consent could not be obtained. However, processing activities were conducted under the legal basis of legitimate interest as scientific research (38). This study was ethically approved by the Ethics Review Board of Tilburg School of Social and Behavioral Sciences (RP858), and data management adhered to GDPR guidelines.

### 2.2 Measures

In the reporting system, staff members followed a structured process to comprehensively document incidents. First, they reported a written summary of the incident, specifying the date of the incident, location (unit), and rating the impact on a scale from 1 (*neutral*) to 5 (*very intrusive*). Staff also had to report the presumed cause of the incident from a list of predefined options: *unknown*, *other patients*, *staff attitude*, *assistance in daily tasks*, *dissatisfaction about treatment*, *medication*, *build-up of tension*, *not identifiable, patient is prohibited of something*, and/or *other*. Then, they indicated who were present during the incident, categorizing them as *patient(s)*, *staff*, *visitors/others*, *nobody*, and *unknown*. They were also required to assess the consequences of the incident, selecting from options such as *none*, *psychological damage*, *physical damage*, *reputational damage*, *property damage*, *danger for staff*, *danger for a patient*, *danger for a third party*, *work absence*, and/or *discomfort*. Both the staff member and the team manager independently rated the severity of the incident on a scale ranging from 0 (*unknown*), 1 (*near-incident*), 2 (*minor incident: affects patient/staff or follow-up processes*), 3 (*major incident: temporary consequences for patient/staff*), 4 (*severe incident: lasting consequences for patient/staff*), to 5 (*fatal incident*). Finally, staff members were asked to categorize the behavior into the following types of aggression: *verbal aggression, verbal menacing*, *physical aggression*, *physical menacing*, *theft*, *aggression related to substance use*, *sexual harassment*/*transgressive behavior*, *possession of prohibited items*, *hostage*, and/or *self-harm.* They also identified the target of the transgressive behavior, which could be *other patient*, *staff*, *self*, *objects*, *nobody/nothing*, and/or *other*.

The patient and staff characteristics obtained were age at the time of the incident and gender (1 = *male* and 2 = *female*). Specifically for staff, their function was included and coded categorically as 1 = *intern/trainee*, 2 = *nursing staff* (including daily care providers such as nurses, sociotherapists, and case managers), 3 = *support staff* (including administrative staff, porters, and technical support), and 4 = *clinical staff* (including treatment providers such as psychologists, psychiatrists, creative therapist, material legal services, systemic therapists, and team managers). For patients, psychopathology was included and coded categorically based on their primary diagnosis with 1 = *psychotic disorder* (including schizophrenic, other psychotic, and bipolar disorders), 2 = *personality disorder*, 3 = *neurobiological developmental disorder* (including ADHD and ASS), 4 = *substance use disorder*, 5 = *mood and/or anxiety disorder* (including depression and PTSD), and 6 = *other diagnoses*. Lasty, the unit where the incident occurred was categorically coded as follows: 1 = *outpatient clinic* (including ambulatory and FACT treatment), 2 = *low security clinic*, 3 = *medium security clinic*, 4 = *high security clinic*, 5 = *clinic with patients with an intellectual disability (ID)*, and 6 = *other facility.*

### 2.3 Statistical analyses

A preregistration (including documented deviations) can be found on OSF (https://osf.io/hbpd7/). A total of 11,487 incidents were reported between 2018 and 2022. However, only 2,194 reported incidents could be accurately matched with both patient and staff data. Manual entry of patient names by staff initially caused inconsistencies, leading to incomplete matches (e.g., only surname/first name or spelling errors). Because this study focused on incidents directed at staff members, only matched data targeting employees were used, resulting in a subsample of 1,184 reported incidents (because of anonymization it is untraceable by how many unique staff members). The data was transferred into Latent GOLD version 6.0 (39) to identify incident classes using LCA. LCA is a model based probabilistic clustering technique, that can be used to estimate classes using observed quantitative indicators (40, 41). The first step is to build a classification model and select the best-fitting model based on the indicators. In the second step, participants are assigned to latent classes based on probabilities. In the third step, the resulting classes are evaluated in relation to external variables. This methodology allows for a quantitative, data-driven classification process and, compared to one-step approaches, adjusts for biases that can affect classification errors (41).

Due to low prevalence of some dichotomous variables in the dataset, which could complicate clustering, only variables exceeding 2% were included. Hence, the LCA was conducted based on 21 indicators. These indicators encompassed impact (ordinal ranging from 1-5), severity independently assessed by both the employee and the team manager (both ordinal ranging from 0-5), presumed causes of the incident (6 dichotomous variables), types of aggression (4 dichotomous variables), and reported consequences of the incident (8 dichotomous variables). Model selection relied on Bayesian information criterion (BIC), Akaike information criterion (AIC), and the modified AIC (AIC3). The model with the lowest value was chosen. Given that AIC tends to overfit while BIC tends to underfit and favors parsimonious (42), more weight was given to BIC in the model selection process. In the next step, the resulting incident clusters were further profiled by comparing characteristics of staff and patients using a bias-adjusted step-three analysis (41). Staff characteristics encompassed age (continuous), gender (nominal: male or female), and professional function (nominal). Patient characteristics included age (continuous), gender (nominal: male or female), and psychopathology (nominal). In addition, incident classes were compared based on the unit where the incident occurred (nominal). Lastly, an example of the written description of incidents was added per class.

## 3 Results

### 3.1 Descriptive statistics

In appendix **Supplementary Table A1**, missing datapoints for each variable are summarized for the selected subsample (*n* = 1,184) and the excluded incidents (*n* = 10,303). In the appendix (**Supplementary Tables A2**–**A4**) an overview is presented on descriptive statistics compared between the final subsample and the excluded incidents. In the final subsample, no missing values were found for the following variables: impact, severity assessed by staff, type of aggression, target, staff age, staff gender, staff function, patient age, and patient gender. Severity assessed by the team manager had 88.6% missing values, (presumed) causes had 20.1%, and consequences, patient diagnosis, and unit each showed less than 5%. In appendix **Supplementary Table A5**, incident rates per year can be found.

### 3.2 Decision on the number of latent classes

Models with one to ten classes were estimated for the subsample of 1,184 incidents. Initial estimation showed substantial bivariate residuals for certain pairs of indicators, showing conceptual similarly and thus large information overlap. For example, the impact and severity assessments were more strongly associated than could be explained by the estimated latent class models. Hence, to account for the large overlap between some of the class indicators, based on the encountered high bivariate residuals, we decided to allow for within-class associations between impact and severity by staff member, impact and severity by team manager, and severity by staff member and team manager. This adjustment reduced the sum of bivariate residuals by 21.0% (in the 6-cluster model). **Table 1** presents the statistics used for class enumeration. The BIC and SABIC values supported the 6-cluster solution, where the values reached a trough. AIC and AIC3 continuously decreased with additional classes, and bootstrap likelihood-ratio test (BLRT) *p*-values remained significant, indicating significant improvement with each added class. Given the emphasis on BIC in model selection, the 6-cluster solution was selected. While classification is not the main purpose, the high Entropy R^2^ value and low estimated proportion of classification errors indicate adequate classification quality in the 6-cluster model.

**Table 1:** Statistics for the deciding on the number of latent classes.

No. of classes	BIC	SABIC	AIC	AIC3	LL	BLRT	Npar	Max. BVR	Class error	Entropy R^2^
1	22101.03	21999.39	21938.58	21970.58	-10937.29		32	763.02	0.00	1.00
2	21039.41	20867.89	20765.28	20819.28	-10328.64	*p* <.001	54	297.59	0.00	0.98
3	20522.60	20281.20	20136.78	20212.78	-9992.39	*p* <.001	76	138.80	0.02	0.95
4	20372.91	20061.63	19875.40	19973.40	-9839.70	*p* <.001	98	78.34	0.09	0.85
5	20323.77	19942.60	19714.57	19834.57	-9737.29	*p* <.001	120	68.85	0.09	0.86
**6**	**20316.52**	**19865.48**	**19595.64**	**19737.64**	**-9655.82**	***p* <.001**	**142**	**52.85**	**0.10**	**0.85**
7	20387.18	19866.26	19554.61	19718.61	-9613.31	*p* <.001	164	28.63	0.14	0.82
8	20460.73	19869.93	19516.47	19702.47	-9572.24	*p* <.001	186	23.15	0.15	0.82
9	20551.23	19890.55	19495.29	19703.29	-9539.65	*p* <.001	208	17.66	0.16	0.81
10	20631.28	19900.72	19463.65	19693.65	-9501.83	*p* <.001	230	18.10	0.15	0.83

Note. BIC = Bayesian information criterion; SABIC = Sample size adjusted BIC; AIC = Akaike information criterion; LL = LogLikelihood; BLRT = bootstrap likelihood ratio test; Npar = number of parameters; BVR = bivariate residuals. The selected model is presented in bold.

### 3.3 Description of the six latent classes

**Figure 1:**
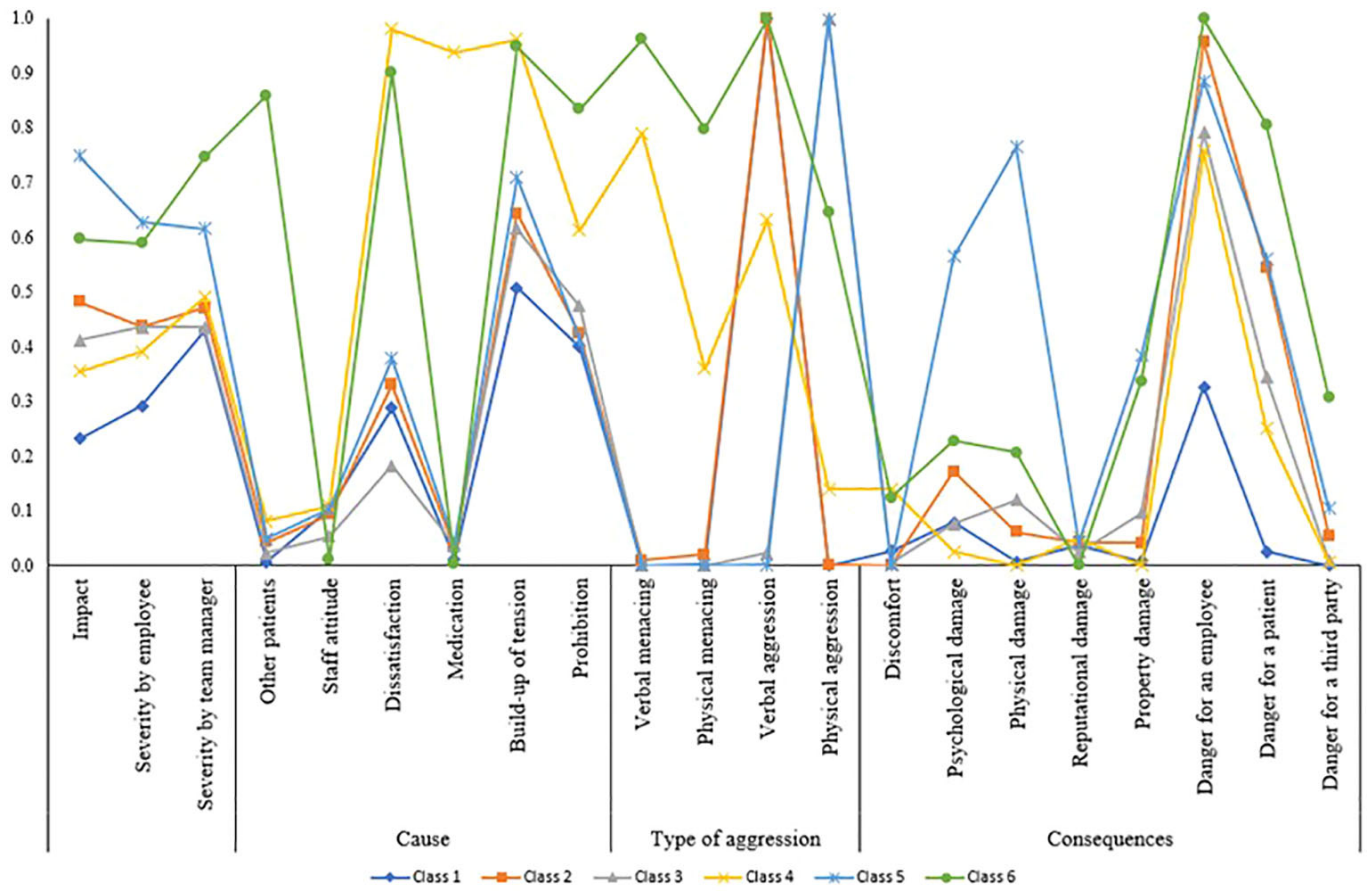
*N* = 1,184. Class 1 (*n* = 368): verbal aggression with low impact; class 2 (*n* = 336): verbal aggression with medium impact; class 3 (*n* = 240): physical aggression with medium impact); class 4 (*n* = 133): verbal menacing and aggression with medium impact; class 5 (*n* = 59): physical aggression with high impact; and class 6 (*n* = 48): verbal and physical menacing and aggression with high impact.

The resulting latent classes are shown in **Table 2** and illustrated in **Figure 1**. The classes are labelled with names about the type of aggression and impact, for reasons of saliency, but these labels are not capturing the full extent of the resulting classes (43). The first class (*verbal aggression with low impact*; *n* = 368) consists of incidents with the lowest impact on employees, primarily characterized by verbal aggression. An example of an incident reported within this class is: “*A patient is absent during lunch and staff confronts him about this when he returns. The patient responds hostile by calling staff a liar. He raised his voice and started scolding*”. The second class (*verbal aggression with medium impact*; *n* = 336), also predominantly includes verbal aggression, though with higher impact and more pronounced consequences for the employees. For instance, “*A staff member could not come to a scheduled appointment with a patient, the patient calls her and leaves a message with insults and a warning that if they will come to the appointment anyway, the patient will put the staff members on fire*”. The third class (*physical aggression with medium impact*; *n =* 240) consists of incidents with a similar severity—assessed by the employees—as the second class but with mostly physical aggressive types of incidents. For example, “*A patient refuses to hand in the phone of the unit after usage. Staff confronts the patient, and the patient spits in the staff members face*”.

**Table 2:** Class-specific probabilities and means of the indicators.

	Class 1 (31.3%)	Class 2 (28.5%)	Class 3 (19.5%)	Class 4 (11.0%)	Class 5 (5.5%)	Class 6 (4.2%)	Wald	R^2^
Ordinal indicators	*M (SE)*	*M (SE)*	*M (SE)*	*M (SE)*	*M (SE)*	*M (SE)*		
Impact (range = 1-5)	1.93 (.16)	2.93 (.21)	2.65 (.19)	2.42 (.24)	3.99 (.27)	3.39 (.40)	50.74^***^	.26
Severity (range = 0-5)								
by employee	1.46 (.13)	2.19 (.14)	2.18 (.13)	1.95 (.19)	3.14 (.17)	2.94 (.24)	63.97^***^	.27
by teammanager	2.15 (.11)	2.35 (.10)	2.18 (.16)	2.45 (.68)	3.08 (.15)	3.73 (.44)	6.93	.25
Dichotomous indicators	*P (SE)*	*P (SE)*	*P (SE)*	*P (SE)*	*P (SE)*	*P (SE)*		
(Presumed) cause								
Other patients	.01 (.01)	.04 (.01)	.02 (.01)	.08 (.08)	.05 (.03)	.86 (.33)	10.98	.45
Staff attitude	.10 (.02)	.09 (.02)	.05 (.02)	.11 (.12)	.10 (.04)	.01 (.10)	3.93	.01
Dissatisfaction	.29 (.03)	.33 (.03)	.18 (.03)	.98 (.05)	.38 (.07)	.90 (.27)	17.39^**^	.26
Medication	.01 (.01)	.03 (.01)	.04 (.02)	.94 (.15)	.04 (.03)	.00 (.06)	8.11	.75
Build-up of tension	.51 (.03)	.64 (.03)	.62 (.03)	.96 (.12)	.71 (.07)	.95 (.19)	13.46^*^	.09
Prohibition of something	.40 (.03)	.42 (.03)	.47 (.04)	.61 (.18)	.41 (.07)	.83 (.46)	3.98	.04
Type of aggression								
Verbal menacing	.00 (.00)	.01 (.02)	.00 (.00)	.79 (05)	.00 (.00)	.96 (.04)	16.41^**^	.80
Physical menacing	.00 (.00)	.02 (.01)	.00 (.00)	.36 (.05)	.00 (.00)	.80 (.07)	77.65^***^	.48
Verbal aggression	1.00 (.00)	1.00 (.00)	.02 (.02)	.63 (.06)	.00 (.01)	1.00 (.01)	47.17^***^	.85
Physical aggression	.00 (.00)	.00 (.01)	1.00 (.00)	.14 (.04)	1.00 (.01)	.64 (.10)	39.57^***^	.89
Consequences								
Discomfort	.03 (.01)	.00 (.00)	.00 (.01)	.14 (.04)	.00 (.00)	.12 (.06)	22.08^***^	.07
Psychological damage	.08 (.02)	.17 (.02)	.08 (.02)	.03 (.02)	.56 (.08)	.23 (.07)	64.54^***^	.12
Physical damage	.01 (.01)	.06 (.01)	.12 (.03)	.00 (.00)	.76 (.08)	.21 (.07)	78.00^***^	.34
Reputational damage	.04 (.01)	.04 (.01)	.02 (.01)	.05 (.02)	.04 (.03)	.00 (.00)	1.67	.00
Property damage	.01 (.01)	.04 (.01)	.10 (.02)	.00 (.00)	.38 (.07)	.34 (.09)	75.60^***^	.17
Danger for employee	.33 (.04)	.96 (.02)	.79 (.03)	.76 (.05)	.89 (.05)	1.00 (.01)	113.29^***^	.34
Danger for patient	.03 (.02)	.54 (.04)	.34 (.03)	.25 (.05)	.56 (.07)	.81 (.09)	68.65^***^	.25
Danger for third party	.00 (.00)	.05 (.01)	.01 (.01)	.01 (.03)	.10 (.04)	.31 (.07)	31.96^***^	.11

*N* = 1,184. For the dichotomous indicators the posterior class membership probability (P) is presented. For the ordinal indicators, the mean per class is given (M). Wald test is used to test if the indicator discriminates between the clusters. R^2^ indicates the communality of the indicator.

^*^p <.05, ^**^p <.01, and ^***^p <.001.

The fourth class (*verbal menacing and aggression with medium impact*; *n* = 133) includes incidents characterized by verbal aggression and menacing incidents with medium impact, relatively more often caused by dissatisfaction, medication, build-up of tension and/or prohibition of something by the employees. An example of an incident is “*After receiving his medication, the patient spits it out in the trash and refuses to take new medications. Staff confronts him and the patient becomes verbally aggressive by insulting and threatening to hit the staff*”. The fifth class (*physical aggression with high impact*; *n* = 59) contains the most impactful and severe (assessed by the employees) incidents with physical and/or psychological injuries as consequences. For instance, “*A patient refuses to hand in a lighter, the patient goes to his room and on his way grabs a knife in the kitchen. Staff manages to confiscate the knife. Then the patient throws the lighter to the staff and insults her. The patient starts a physical fight with staff and is separated*”. Finally, the sixth class (*verbal and physical menacing and aggression with high impact*; *n* = 48) includes incidents with mixed types of aggression and is assessed as the most severe by team managers. These incidents are often triggered by other patients and factors like dissatisfaction, build-up of tension and the patient being prohibited of something. Moreover, incidents in the sixth class are rated highest on consequences of danger for both employees and patients. For example, “*A patient who is locked in his room threatens to pull a staff member’s hair and makes a gun gesture with his hands aiming at staff making gun sounds. He keeps on shouting the name of the staff member and is physically aggressive towards the doors and windows*”.

### 3.4 Profiling the classes with external variables (step-three analysis)

**Figure 2:**
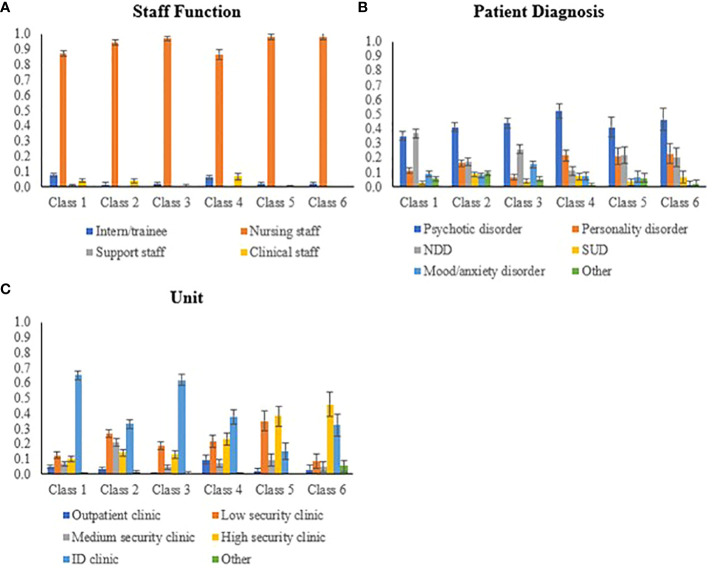
Graphical comparison between classes on staff function **(A)**, patient psychopathology **(B)**, and units **(C)**.

**Figure 3:**
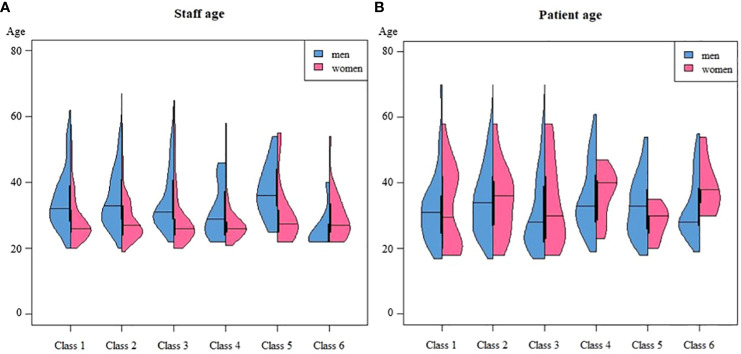
Graphical comparison between classes on staff **(A)** and patient **(B)** age, disaggregated by gender.

After saving the classification, we investigated differences in staff, patient, and unit/clinic characteristics among the identified classes using a bias-adjusted step-three analysis. Regarding staff characteristics (**Table 3**; **Figure 2A**), incidents overall primarily target nursing staff (including daily care providers). Exceptions include incidents in classes 1, 2 and 4, which also involve clinical staff. Classes 1 and 4 additionally include incidents targeting interns/trainees. Female employees are more frequently targeted in all classes, except class 6. Besides, staff age was generally higher in classes 1 and 5 compared to classes 2 and 4. When investigating age differences between male and female staff (**Figure 3A**), additional differences emerge across classes (considering sample size dependency). For instance, male staff was generally older than female staff in classes 1 to 5, but younger in class 6.

**Table 3:** Class-specific probabilities and means of the external variables.

	Class 1 (30.5%)	Class 2 (28.5%)	Class 3 (19.6%)	Class 4 (11.8%)	Class 5 (5.5%)	Class 6 (4.2%)	Wald
Numeric variables	*M (SE)*	*M (SE)*	*M (SE)*	*M (SE)*	*M (SE)*	*M (SE)*	
Staff age	31.55 (0.47)	29.86 (0.43)	30.74 (0.65)	28.71 (0.70)	32.71 (1.43)	27.21 (1.28)	21.60^***^
Patient age	31.67 (0.61)	35.31 (0.68)	31.94 (0.89)	35.90 (0.94)	33.00 (1.41)	32.56 (1.31)	18.92^**^
Nominal variables	*P (SE)*	*P (SE)*	*P (SE)*	*P (SE)*	*P (SE)*	*P (SE)*	
Staff gender							22.96^***^
Male	.34 (.03)	.22 (.03)	.34 (.03)	.33 (.05)	.29 (.06)	.58 (.08)	
Female	.66 (.03)	.78 (.03)	.66 (.03)	.67 (.05)	.71 (.06)	.42 (.08)	
Patient gender							29.47^***^
Male	.85 (.02)	.89 (.02)	.72 (.03)	.89 (.03)	.92 (.04)	.87 (.05)	
Female	.15 (.02)	.11 (.02)	.28 (.03)	.11 (.03)	.08 (.04)	.13 (.05)	
Staff function							123.95^***^
Intern/trainee	.08 (.02)	.02 (.01)	.02 (.01)	.07 (.02)	.02 (.02)	.02 (.02)	
Nursing staff	.87 (.02)	.94 (.02)	.97 (.01)	.87 (.03)	.98 (.02)	.98 (.02)	
Support staff	.01 (.01)	.00 (.00)	.00 (.00)	.00 (.00)	.00 (.00)	.00 (.00)	
Clinical staff	.04 (.01)	.04 (.01)	.01 (.01)	.07 (.02)	.00 (.00)	.00 (.00)	
Patient diagnosis							74.22^***^
Psychotic disorder	.35 (.03)	.41 (.03)	.44 (.04)	.52 (.05)	.41 (.07)	.46 (.08)	
Personality disorder	.11 (.02)	.16 (.02)	.07 (.02)	.21 (.04)	.21 (.06)	.23 (.07)	
NDD	.37 (.03)	.17 (.02)	.26 (.03)	.11 (.03)	.21 (.06)	.20 (.06)	
SUD	.03 (.01)	.09 (.02)	.04 (.01)	.07 (.02)	.03 (.02)	.07 (.04)	
Mood/anxiety disorder	.09 (.02)	.08 (.02)	.15 (.03)	.07 (.03)	.07 (.04)	.02 (.02)	
Other	.05 (.01)	.09 (.02)	.05 (.02)	.01 (.01)	.06 (.03)	.02 (.02)	
Unit							157.42^***^
Outpatient clinic	.05 (.01)	.03 (.01)	.01 (.01)	.10 (.03)	.02 (.02)	.03 (.03)	
Low security clinic	.12 (.02)	.27 (.03)	.19 (.03)	.22 (.04)	.35 (.07)	.09 (.05)	
Medium security clinic	.07 (.02)	.21 (.02)	.05 (.02)	.07 (.03)	.09 (.04)	.05 (.03)	
High security clinic	.10 (.02)	.14 (.02)	.13 (.02)	.23 (.04)	.38 (.07)	.46 (.08)	
ID clinic	.65 (.03)	.33 (.03)	.62 (.03)	.38 (.05)	.15 (.06)	.32 (.07)	
Other	.01 (.01)	.02 (.01)	.01 (.01)	.01 (.01)	.00 (.00)	.05 (.03)	

For the nominal variables the class-specific probability (P) is presented. For the numeric variables, the class-specific mean is given (M). Wald statistic tests whether the differences between the classes is significant. NDD = Neurodevelopmental disorder; SUD = Substance use disorder; ID clinic = clinic for patients with intellectual disabilities.

^*^p <.05, ^**^p <.01, and ^***^p <.001.

Overall, patients involved in the incidents were mostly diagnosed with a psychotic disorder (see **Table 3**; **Figure 2B**), with some variations among classes. In class 1, patients with psychotic or neurodevelopmental disorders were most prevalent. Class 2 displayed a more varied profile. Class 3 included a higher proportion of patients with mood/anxiety disorders compared to other classes. In classes 4, 5, and 6, we found relatively more patients with personality disorders alongside psychotic disorders. The age of patients in classes 2 and 4 was slightly higher compared to other classes. Specifically in classes 4 and 6, female patients were, on average, older than male patients (**Figure 3B**). Across all classes, there were more male patients than female patients, although class 3 showed a relatively higher number of female patients compared to other classes.

Regarding context characteristics (**Table 3**; **Figure 2C**), incidents in classes 1 and 3 mainly occur in clinics with patients with intellectual disabilities (ID clinics). Classes 2 and 4 display a more varied distribution, with higher occurrence rates in ID clinics but also extending across low and high security clinics. Incidents in class 5 mainly take place in low and high security clinics, while incidents in class 6 are primarily observed in high security, occasionally extending to ID clinics. These findings imply that incidents with relatively higher impact (classes 5 and 6) are more common in high security clinics, while incidents with lower to medium impact tend to occur more frequently in ID clinics.

## 4 Discussion

This study aimed to identify clusters of transgressive incidents by patients targeting staff in forensic psychiatric healthcare. This was done based on incident characteristics, such as impact, severity, (presumed) cause, type of aggression, and consequences. Subsequently, the study investigated whether these clusters varied for staff, patient, and context characteristics. Six classes of incidents were identified: 1) *verbal aggression with low impact*; 2) *verbal aggression with medium impact*; 3) *physical aggression with medium impact*; 4) *verbal menacing and aggression with medium impact*; 5) *physical aggression with high impact*; and 6) *verbal and physical menacing and aggression with high impact*. Most incidents resulted from a build-up of tension, which is in line with previous findings on stress-related physiological measures (such as skin conductance and heart rate) preceding inpatient aggression (44). Specific causes of incidents varied among classes (e.g., *medication* in class 4 and *other patients* in class 6). The consequences of almost all types of incidents (except class 1) mainly included danger to staff, and, to a lesser extent, patients (counting the initiator and/or fellow patients). For class 5 particularly, staff members reported psychological and physical damage more frequently as a consequence of the transgressive incidents. This is in accordance with the increased effects of physical (vs. verbal) aggression and negative psychological impact in nurses (45).

Nursing staff was predominantly targeted (92.1%) and evenly distributed across classes. This can be attributed to their extensive interaction (both in time and type of contact) with patients compared to interns/trainees, clinical, and support staff, thus increasing their vulnerability to violence (46). Another explanation, in accordance with Peternelj-Taylor and Yonge (47), could be that nursing staff may face heightened risks of experiencing a “seductive pull of helping” or “compulsive caring” leading to over-involvement and possible boundary confusion (46). Seductive pull can refer to a phenomenon where a therapist unintentionally and unconsciously exerts an emotional dynamic that crosses professional boundaries, drawing the patient towards the therapist. Recognizing and managing these dynamics is essential in maintaining ethical therapeutic practices (48).

Against expectations (29), female staff was most often targeted in classes 1-5, possibly due to the relatively higher number of women (76% vs. 34% men) working in mental healthcare (49). This aligns with the finding that female and young staff (age < 40) is more vulnerable to non-physical forms of aggression (28). However, for incidents in class 6 (*verbal and physical menacing and aggression with high impact*), relatively more male staff was targeted. Notably, in class 6, the mean age of male staff was the lowest among all classes, consistent with the general trend where younger and less experienced staff (vs. older and more experienced) is more susceptible to workplace violence (26). On the contrary, the mean age of male staff was relatively higher in class 5 (*physical aggression with high impact*), indicating that besides impact and severity, the type of aggression (i.e., physical vs. verbal) may also play a role. A previously given explanation for the higher proportion of targeted male (vs. female) staff could be that female staff are more adept at preventing and deflecting severe physical violence, or male staff is more frequently called upon in situations of extreme aggression (28).

Moreover, individual characteristics of staff members might influence the occurrence and nature of incidents in multiple ways. For instance, the stress-reactivity in forensic workers can influence the risk of physically aggressive incidents from patients (29). This can be attributed to past traumatic experiences, but is also dependent on personality traits (50). High scores on openness to experience can be connected to better stress-regulation (51), and an active coping style might buffer the negative effects of incidents on staff well-being (52). On the contrary, neuroticism has been linked to poorer stress-regulation and an increased risk of burn-out in nurses (53). Specifically, neuroticism has been negatively linked to forensic vigilance, which involves anticipating possible escalation of situations which can be interpreted as a crucial skill for forensic staff (54). Hence, earlier (traumatic) experiences, personality traits, and coping skills of the staff member might play a role in handling with situations leading up to and during transgressive incidents.

For the patient characteristics, consistent with expectations (31), patients were mostly male and primarily diagnosed with a psychotic disorder. This can be explained by the relatively higher number of male patients (92% vs. 8% female) in Dutch forensic psychiatric healthcare (55). However, across the six classes, there was a relatively higher number of incidents caused by women in class 3 (*physical aggression with medium impact*), partially aligning with previous findings indicating that female patients relatively cause more physically aggressive incidents (36, 37). This was previously explained by complex psychopathology (e.g., borderline personality disorder, history of victimization, and homicide/arson; 36)), but the current findings suggest a higher prevalence of mood and anxiety disorders in class 3, which are generally more prevalent in women than men (56).

Regarding context characteristics, the finding that more impactful incidents (i.e., classes 5 and 6) occurred more frequently in high security clinics can have multiple explanations. In the Netherlands, forensic patients are placed in clinics based on the required security level imposed by court following the RNR principles (14), meaning patients with higher risks of violent behavior and more severe psychopathology are placed in more secured clinics. In general, there is more personal boundary setting in highly secured forensic units, which directly correlates with an increase in patient aggression, regardless of psychopathology (57). Moreover, in the current study, clinics for patients with intellectual disabilities (ID clinics) were grouped despite varying security-levels, possibly explaining the high occurrence of all incidents in ID clinics.

Then, when examining the reporting behavior regarding incidents in general, it is striking that team managers generally assessed severity higher than staff themselves, though with a high percentage of missing information in severity assessment of incidents by team managers. This could be explained by a response bias, where team managers might be more inclined to assess incidents as more severe in serious cases. Alternatively, staff members might underestimate the severity of incidents due to insufficient reflection or self-awareness skills essential for boundary management (58). Staff members could also use downregulation techniques as coping mechanisms to mitigate the impact and consequences of incidents, as is seen in empathic responses in hospital nurses to downregulate inflicting pain (59). This behavior reflects cognitive dissonance, internal discomfort arising from conflicting cognitive dissonance needing reconciliation (60). Staff might minimize the impact of the incident using cognitive dissonance to preserve self-concept and social status among colleagues. Especially the link with low self-esteem can explain why an individual stays in an abusive context despite conflicting beliefs about themselves as a consequence of the conflict, as is often observed in victims of intimate partner violence (61). Nevertheless, incongruent severity assessments in this study confirms the subjectivity of incident severity, not only between patients and staff (with differing views on incident causes; 62), but also among professionals. This underscores the need for individually tailored prevention approaches.

### 4.1 Strengths, limitations, and future recommendations

This study uses real-life data, providing ecologically valid insights with a high number of reported incidents, thereby demonstrating sufficient power and highly relevant findings. However, the downside of this real-life data is the presence of missing information. As the data collection was not primarily for research purposes, incident categorization may be somewhat subjective, with staff members possibly interpreting response options differently due to time constraints and high-pressure work environments, which can lead to potential bias in incident reports. Future studies could benefit from using instruments with robust psychometric qualities, such as the Modified Overt Aggression Scale (MOAS+; 63). Moreover, there are a lot of possible individual characteristics that can influence the impact of incidents on staff members, such as stress-reactivity (e.g., stress-reactivity; 29), cognitive dissonance (60, 64), and personality and coping strategies in general. These characteristics could also influence the reporting style or frequency of reporting incidents, but due to data anonymization, controlling for them was not possible.

Although these six classes show distinguishable incidents with differing causes and consequences, which can be linked to different patient, staff, and context characteristics, these analyses do not inform why incidents are occurring. Information about the motives and goals of patients to show aggression could not be derived, despite suggestions that understanding these motives and goals can help in managing incidents (9, 65). When attempting to explain the occurrence of these transgressive incidents using the General Aggression Model (GAM; 11), incidents were clustered based on the input and output of aggressive behavior, but the cognitive, affective, and arousal routes/mechanisms remain unknown. Future research should explore the underlying motives driving transgressive behavior in patients, while considering the heterogeneity and complex interplay of incidents, patients, staff, and context. In addition, information about the consequences of transgressive behavior for the inflictor (i.e., patient) is unknown. It can be presumed that patients who cause transgressive incidents have a prolonged treatment duration, but there have been no studies to explore the (in)direct (psychological) consequences for patients (6). Hence, future research could investigate the consequences on patient well-being and treatment processes on a more qualitative level.

Furthermore, although the unit where the incident occurred was included in the current study, there are many other contextual factors that influence incidents, staff members, and patients. For instance, a positive working relationship with colleagues has been identified as a protective factor for staff dealing with patient transgressive behavior (30, 58). Organizational factors can also promote a feeling of safety, which potentially increases confidence in managing aggression (8). It is recommended to investigate organizational and context factors at the macro-level (e.g., across institutions) in boundary management within forensic psychiatric healthcare. Future research could investigate how specific contextual factors, such as treatment programs and facility infrastructure within institutions could contribute to the occurrence and severity of incidents. Moreover, long-term effects on the psychological well-being and job satisfaction of staff could be investigated to understand repeated exposure to aggression, repeated victimization, and their impact on staff turnover and burnout rates.

### 4.2 Conclusion

Our study indicates that different types of transgressive incidents occur among different groups of patients, staff, and units. Our findings underscore the need for a more tailored approach to prevent transgressive incidents in forensic psychiatric healthcare. Additionally, there is a need for research into the underlying motives and goals driving transgressive behavior in patients and into factors contributing to the impact of incidents on the well-being of staff members and patients.

## Data availability statement

The original contributions presented in the study are included in the article/supplementary materials, further inquiries can be directed to the corresponding author/s.

## Ethics statement

The studies involving humans were approved by Ethics Review Board of Tilburg School of Social and Behavioral Sciences (RP858). The studies were conducted in accordance with the local legislation and institutional requirements. Written informed consent for participation was not required from the participants or the participants’ legal guardians/next of kin because pre-collected data were used, hence informed consent could not be obtained in accordance with the GDPR assessment. Processing activities were conducted under the legal basis of legitimate interest as scientific research.

## Author contributions

IF: Conceptualization, Project administration, Writing – original draft. EM: Conceptualization, Supervision, Writing – review & editing. JV: Methodology, Writing – review & editing. SB: Conceptualization, Supervision, Writing – review & editing.
