# Supplementary material for: Transgressive incidents targeted on staff in forensic psychiatric healthcare: a latent class analysis
Source: Front Psychiatry. 2024 May 20;15:1394535. doi: 10.3389/fpsyt.2024.1394535 (PMC11145633; doi:10.3389/fpsyt.2024.1394535)
Supplement: Supplementary file 1 [file DataSheet_1.pdf]

frontiers

Appendix

Table A1

Overview of missing data for each variable

	Subsample (n = 1,184)		Excluded incidents (n = 10,303)	
	n	%	n	%
Missings				
Impact	0	0.0%	0	0.0%
Severity (by staff)	0	0.0%	0	0.0%
Severity (by team manager)	1,049	88.6%	9,069	88.0%
(Presumed) causes	238	20.1%	5,581	54.2%
Type of aggression	0	0.0%	1	0.0%
Target	0	0.0%	3,073	29.8%
Consequences	7	0.6%	91	8.8%
Staff age	0	0.0%	3,765	36.5%
Staff gender	0	0.0%	3.765	36.5%
Staff function	0	0.0%	3.232	31.4%
Patient age	0	0.0%	9,050	87.8%
Patient gender	0	0.0%	9,050	87.8%
Patient diagnosis	42	3.5%	9,102	88.3%
Unit	99	1.0%	6	0.5%

Note. N = 11,487. Subsample (n = 1,184) was selected based on matched incidents (i.e., information about both staff member and patient was available) and targeted at employee(s).

Table A2

Comparison between selected subsample and excluded incidents for ordinal variables

		Subsample (n = 1,184)	Excluded incidents (n = 10,303)	Group differences
	Range	M (SD)	M (SD)	
Impact	1–5	2.58 (1.09)	2.18 (1.09)	$t(11485) = 12.02^{***}$
Severity (by staff)	0–5	2.01 (0.89)	1.95 (0.86)	$t(11485) = 2.60^{**}$
Severity (by team manager)	0–5	2.37 (0.69)	2.59 (0.73)	$t(1367) = -3.38^{***}$
Staff age	19–67	30.54 (8.24)	33.85 (11.12)	$t(2051.36) = -11.98^{***}$
Patient age	17–70	33.29 (10.78)	31.60 (11.60)	$t(2435) = 3.71^{***}$

Note. N = 11,487. Subsample (n = 1,184) was selected based on matched incidents (i.e., information about both staff member and patient was available) and targeted at employee(s).

$^{*}p < .05$, $^{**}p < .01$, and $^{***}p < .001$.

Table A3

Comparison between selected subsample and excluded incidents for nominal variables

	Subsample (n = 1,184)		Excluded incidents (n = 10,303)		Group differences
	n	%	n	%	
(Presumed) cause					
Unknown[c]	31	3.3%	205	4.3%	$\chi^2 = 2.24$
Other patients	26	2.7%	582	12.3%	$\chi^2 = 75.48^{***}$
Staff attitude	84	8.9%	306	6.5%	$\chi^2 = 7.08^{**}$
Assistance in daily tasks[p]	8	0.8%	25	0.5%	$\chi^2 = 1.36$
Dissatisfaction about treatment	278	29.4%	1,129	23.9%	$\chi^2 = 12.67^{***}$
Medication	36	3.8%	103	2.2%	$\chi^2 = 8.69^{**}$
Build-up of tension	565	59.7%	2,798	59.3%	$\chi^2 = 0.07$
Prohibition of something	406	42.9%	1,419	30.1%	$\chi^2 = 59.77^{***}$
Not identifiable[c]	88	9.3%	336	7.1%	$\chi^2 = 5.45^{*}$
Other[c]	119	12.6%	578	12.2%	$\chi^2 = 0.08$
Type of aggression					
Verbal menacing	154	13.0%	1,192	11.6%	$\chi^2 = 2.12$
Physical menacing	94	7.9%	725	7.0%	$\chi^2 = 1.30$
Verbal aggression	845	71.4%	4,656	45%	$\chi^2 = 295.94^{***}$
Physical aggression	347	29.3%	2,751	26.7%	$\chi^2 = 3.66$
Possession of prohibited items[p]	1	0.1%	267	2.6%	$\chi^2 = 29.30^{***}$
Substance use related[p]	2	0.2%	883	8.6%	$\chi^2 = 105.42^{***}$
Theft[p]	0	0.0%	57	0.6%	$\chi^2 = 6.58^{*}$
Sexual transgressive behavior[p]	9	0.8%	548	5.3%	$\chi^2 = 47.84^{***}$
Self-harm[p]	2	0.2%	1,552	15.1%	$\chi^2 = 201.42^{***}$
Hostage[p]	0	0.0%	11	0.1%	$\chi^2 = 1.27$
Target					
Nobody/nothing[s]	7	0.6%	300	4.1%	$\chi^2 = 36.64^{***}$
Other patient(s)[s]	75	6.3%	1,367	18.9%	$\chi^2 = 113.25^{***}$
Employee[s]	1,184	100.0%	4,424	61.2%	$\chi^2 = 689.44^{***}$
Objects[s]	152	12.8%	1,691	23.4%	$\chi^2 = 66.21^{***}$
Self[s]	57	4.8%	479	6.6%	$\chi^2 = 5.59^{*}$
Other[s]	24	2.0%	414	5.7%	$\chi^2 = 28.21^{***}$
Consequences					
None[c]	260	22.1%	2,347	23.0%	$\chi^2 = 0.48$

Discomfort	35	3.0%	650	6.4%	$\chi^2 = 21.47^{***}$
Psychological damage	155	13.2%	1,260	12.3%	$\chi^2 = 0.67$
Physical damage	110	9.3%	1,870	18.3%	$\chi^2 = 59.07^{***}$
Reputational damage	43	3.7%	406	4.0%	$\chi^2 = 0.29$
Property damage	80	6.8%	943	9.2%	$\chi^2 = 7.67^{**}$
Danger for employee	827	70.3%	4,660	45.6%	$\chi^2 = 256.45^{***}$
Danger for patient	379	32.2%	3,704	36.3%	$\chi^2 = 7.60^{**}$
Danger for third party	43	3.7%	420	4.1%	$\chi^2 = 0.571$
Work absence^{p}	6	0.5%	41	0.4%	$\chi^2 = 0.301$

Note. Overview of all variables used to estimate classes in the Latent Class Analysis. Variables presented in grey were excluded based on ^{p}prevalence (< 2.0% in the subsample), ^{c}classification (answers on these categories conceptually inferred with answers on other categories, which could lead to overclassification), and ^{s}select (the target variable was used to select the subsample).

$^{*}p < .05$, $^{**}p < .01$, and $^{***}p < .001$.

Table A4

Comparison between selected subsample and excluded incidents for nominal (step 3) variables

	Subsample (n = 1,184)		Excluded incidents (n = 10,303)		Group differences
	n	%	n	%	
Staff gender					$\chi^2 = 1.35$
Male	366	30.9%	1,912	29.2%	
Female	818	69.1%	4,627	70.8%	
Patient gender					$\chi^2 = 26.07^{***}$
Male	1,000	84.5%	955	76.2%	
Female	184	15.5%	298	23.8%	
Staff function					$\chi^2 = 14.75^{**}$
Intern/trainee	50	4.2%	253	3.6%	
Nursing staff	1,091	92.1%	6,417	90.9%	
Support staff	5	0.4%	120	1.7%	
Clinical staff	38	3.2%	257	3.6%	
Patient diagnosis					$\chi^2 = 51.26^{***}$
Psychotic disorder	469	41.1%	418	34.8%	
Personality disorder	161	14.1%	149	12.4%	
NDD	280	24.5%	326	27.1%	
SUD	60	5.3%	106	8.8%	
Mood/anxiety disorder	105	9.2%	174	14.5%	
Other	67	5.9%	28	2.3%	
Unit					$\chi^2 = 716.36^{***}$
Outpatient clinic	46	3.9%	601	5.9%	
Low security clinic	234	19.9%	2,040	20.0%	
Medium security clinic	119	10.1%	266	2.6%	
High security clinic	197	16.7%	348	3.4%	
ID clinic	564	47.9%	5,457	53.5%	
Other	18	1.5%	1,492	14.6%	

Note. NDD = neurodevelopmental disorder; SUD = substance use disorder; ID = Intellectual disabilities

$^{*}p < .05$, $^{**}p < .01$, and $^{***}p < .001$.

Table A5

Number of reported incidents per year

	Total sample (N = 11,487)		Final subsample (n = 1,184)	
Year	N	%	N	%
2018[a]	77	0.7%	10	0.8%
2019	2,532	22.0%	325	27.4%
2020	2,644	23.0%	352	29.7%
2021	2,943	25.6%	316	26.7%
2022	3,292	28.7%	181	15.3%

Note. Considering that the dataset encompasses incidents during the covid-19 pandemic, we inspected incident rates per year to control for possible pandemic-related influences. Although many factors may influence incident rates (e.g., number of patients), the data does not indicate a significant increase in incidents attributable to the covid-19 pandemic.

[a]The relatively lower number of incidents in 2018 can be explained by operational developments in the incident reporting system.
